# Promoting rational antibiotic use in Turkey and among Turkish migrants in Europe – implications of a qualitative study in four countries

**DOI:** 10.1186/s12992-020-00637-5

**Published:** 2020-11-11

**Authors:** R. Westerling, A. Daryani, O. Gershuni, K. Czabanowska, H. Brand, F. Erdsiek, T. Aksakal, S. Uner, O. Karadag Caman, H. Ozcebe, P. Brzoska

**Affiliations:** 1 Department of Public Health and Caring Sciences, Uppsala University, Uppsala, Sweden; 2 Department of International Health, Maastricht University, FHML, CAPHRI, Maastricht, Netherlands; 3 Faculty of Health, School of Medicine, Health Services Research, Witten/Herdecke University, Alfred-Herrhausen-Straße 50, D-58448 Witten, Germany; 4 Faculty of Behavioral and Social Sciences, Epidemiology Unit, Chemnitz University of Technology, Chemnitz, Germany; 5 Hacettepe University, Institute of Public Health, Ankara, Turkey; 6 Faculty of Medicine Department of Public Health, Lokman Hekim University, Ankara, Turkey; 7 Center for Sustainable Development, Columbia University, Earth Institute, New York, NY USA; 8 Faculty of Medicine Department of Public, Hacettepe University, Ankara, Turkey

**Keywords:** Antimicrobial resistance, Antibiotics, Health system, Health policy, Migrants, Turkey, Sweden, Germany, The Netherlands, Quality of care

## Abstract

**Background:**

Antimicrobial resistance is considered one of the major threats to global health. The emergence of resistant microorganisms is a consequence of irrational use of antibiotics. In Turkey, the consumption of antibiotics is relatively high and antibiotics are among the most commonly used drugs. However, Turkey has adopted new, more restrictive policies and regulations on antibiotics. In addition, Turkish migrants to EU countries, such as Germany, the Netherlands and Sweden, may encounter health systems that promote a more restrictive and rational antibiotic use.

The objective of this paper was to explore the variation in implemented policies related to rational antibiotic use that citizens in Turkey and Turkish migrants in Germany, the Netherlands and Sweden are subjected to and to discuss the implications for the promotion of rational antibiotic use.

Data were collected through focus groups and individual interviews with citizens, physicians and pharmacists in the four countries. In total, 130 respondents were interviewed. Content analysis was used.

**Results:**

Three relevant themes were identified: Implementation of regulations and recommendations, Access to antibiotics and Need for health communication. Irrational use of antibiotics was reported mainly in Turkey. While it had become less likely to get antibiotics without a prescription, non-prescribed antibiotics remained a problem in Turkey. In the three EU countries, there were also alternative ways of getting antibiotics. Low levels of knowledge about the rational antibiotic use were reported in Turkey, while there were several sources of information on this in the EU countries. Communication with and trust in physicians were considered to be important. There were also system barriers, such as lacking opportunities for physicians to manage care in accordance with current evidence in Turkey and factors limiting access to care in EU countries.

**Conclusions:**

Several fields of importance for promoting rational antibiotic use were identified. There is a need for harmonisation of health-related regulations and policy programmes. Antibiotics should only be available with a prescription. Programmes for rational antibiotic use should be implemented on a broad scale, in medical care, at pharmacies and in the population. Methods for health communication and patient-centred care should be further developed and implemented in this field.

## Background

While the development of antibiotics has been one of the major advances in medicine, the benefits of these drugs are now seriously threatened [1]. Development of antimicrobial resistance (AMR) is in part due to the overuse of antibiotics in the population and environment. Excessive or unnecessary use of antibiotics does not only increase health care expenditure, but can also be detrimental to human health, for instance through unwanted side effects for users and the risk for emergence of resistant microorganisms, which may cause antibiotics to be ineffective against certain microbes [2]. The World Health Organization (WHO) has identified AMR as one of ten major threats to global health [3].

In 2015, the WHO World Health Assembly adopted an action plan on AMR [4]. The plan has five objectives, including to improve awareness and understanding of AMR, to optimise the use of antimicrobial medicine and to ensure sustainable investments for countering AMR. The Assembly urged WHO member states to implement the plan, recognising that it might require adaption to specific contexts in different countries.

In September 2016, the United Nations (UN) held a meeting dedicated to agreeing upon an effective global plan for managing the problem of AMR. The meeting resulted in a high-level political declaration on AMR [5]. Thus, there is a strong political agreement among UN member states to take action against AMR. The UN states that prevention of AMR is an issue of sustainable development. The declaration stresses the need to mobilise resources in all countries and through international and multi-sectorial collaborations to manage the risks of AMR. The UN also highlights the right of access to health facilities, health care professionals, diagnostic and preventive tools, knowledge, education and information as strategies for achieving universal health [5]. In the European Union there are also regulations in the Veterinary Sanitary and Food Safety Area relevant for preventing AMR [6–9].

Despite the prioritisation of managing the AMR threat to public health, surveillance of AMR in Europe shows high levels of AMR in several bacteria strains, as well as ineffective antibiotics. There is variation between different countries and geographical regions, with lower levels of AMR in Northern Europe and higher levels in Southern and Eastern Europe [10]. This pattern also corresponds to the country variations in use of antibiotics within the European Union (EU) [11]. Similar analyses have also been performed for the Eastern European countries, showing even larger variations, with the highest total consumption in Turkey [12].

An EU-supported publication reported different causes of imprudent use of antibiotics in the EU countries [13]. The objectives were to identify key factors that drove the sale and use of antibiotics without a prescription, as well as to study legislation regarding prescription rules for antibiotics. The use of antibiotics without a prescription has been considered an irrational use of antibiotics, due to the lack of medical assessment and guidance [14]. Most antibiotics in the EU were dispensed following prescription. However, the proportion of non-dispensed use increased somewhat from 5 to 7% between 2013 and 2016, even though the sale of non-dispensed antibiotics was illegal in all EU countries. There was a variation between countries, with higher rates in Eastern and Southern Europe [14]. Factors that influenced the use of non-dispensed antibiotics were identified at the patient, health care professional and health care system level, using data from literature reviews and questionnaires. The main issues were found to be knowledge dissemination and patient education, while factors relating to professionals and systems were not fully explored.

In Turkey, the consumption of antibiotics is relatively high and antibiotics have been among the most commonly used drugs for many years [15]. In 2014, the Turkish government introduced a national action plan on rational drug use which prioritised rational use of antibiotics [12, 16]. Fields that were highlighted included international collaboration, policy issues, surveillance and raising awareness. Legislations were changed and, under the new rules, antibiotics must be prescribed by a physician and over-the-counter sales are no longer permitted [16]. Thus, Turkey is in a transition towards new policies and regulations that promote more restrictive antibiotic use. However, little is known about the professionals’ and the public’s experiences of this transition.

There are many migrants from Turkey in various EU countries such as Germany, the Netherlands and Sweden. In these countries, more restrictive regulations and policies have been at hand for a longer time and may be expected to be established to a higher degree within health care, at pharmacies and in general awareness among the population. National action plans were established in these countries in 2000 (Sweden), 2008 (Germany) and 2015 (the Netherlands), respectively [17].

Germany, the Netherlands and Sweden are among the countries with the lowest rates of antibiotic use in the population, as well as the lowest rates of people reporting having taken antibiotics without a prescription [13]. Moving from Turkey to one of these EU countries would also mean moving from one health system to another in regard to how the antibiotic use is managed. How migrants perceive this change has not been explored previously. This study focuses on rational antibiotic use in Turkey and in three EU countries: Germany, the Netherlands and Sweden. We expect these assessments to be an important step in analysing how the implementation of health policies may need to be developed in this field.

## Methods

The aim of this study was two-fold: 1) to explore the variation in implemented policies related to AMR in Germany, the Netherlands, Sweden and Turkey and the perceived access to antibiotics and information on rational antibiotic use among Turkish migrants living in the three EU countries and citizens in Turkey; 2) to discuss the implications of these findings for the promotion of rational antibiotic use.

A qualitative study design was used, based on focus group and in-depth interview data. Data were collected from citizens in Turkey and Turkish migrants in Germany, the Netherlands and Sweden, as well as from physicians and pharmacists in these four countries. A total of 130 respondents were interviewed in the four countries during 2016 [14].

Data from Turkish citizens in Turkey and Turkish migrants in the three EU countries included in this study were collected through three focus groups in Turkey (*n* = 37), two focus groups in Germany (*n* = 11), eight in-depth interviews in the Netherlands (*n* = 8) and four focus groups in Sweden (*n* = 28). The participants were recruited strategically in order to, as far as feasible, get a variation in the gender, ages and educational background of the respondents (Table 1).

**Table 1:** Sociodemographic characteristics of the respondents among the four countries

	Turkey	Germany	The Netherlands	Sweden
***Citizens in Turkey/Turkish migrants in EU countries***				
Gender (number male/females)	12/25	7/4	4/4	8/20
Ages (min-max)	21–50	24–58	23–55	28–70
Educational level (number primary school/secondary school/ higher education)	15/9/13	4/5/2	0/1/7	8/14/6
***Family physicians***				
Gender (number male/females)	7/4	3/1	2/1	0/3
Ages (min-max)	29–53	42–57	44–50	30–54
Professional experience (range in years)	5–29	10–28	9–25	5–24
***Pharmacists***				
Gender (number male/females)	3/6	1/4	1 (5	1/3
Ages (min-max)	26–55	33–56	26–53	40–55
Professional experience (range in years)	3–30	10–31	3–25	10–25

In-depth interviews were performed with professionals (physicians and pharmacists) in all four countries. In Turkey, 11 physicians and 9 pharmacists participated. In the three EU countries, the professionals were strategically selected in order to find those who had contact with migrants from Turkey and to get a variation in the professional experiences of the respondents. A total of 11 physicians and 15 pharmacists from these countries participated (5 physicians and 5 pharmacists in Germany; 3 physicians and 6 pharmacists in the Netherlands; 3 physicians and 4 pharmacists in Sweden).

The interviews were based on interview guides developed by the research team, one for each of the three target groups: 1) citizens in Turkey and Turkish migrants in the three EU countries, 2) physicians and 3) pharmacists. The interview guides were semi-structured and included open-ended questions, all related to antibiotic use, and were translated into national languages.

The guides for interviews with the population in Turkey and Turkish migrants in the three EU countries included questions about attitudes towards and behaviours in regard to the use of antibiotics and information about antibiotics. The physicians were asked about antibiotics prescription (guidelines, habits, etc.), experiences regarding the contact and communication with Turkish citizens in Turkey and Turkish migrants in the three EU countries, and what facilitated or limited such communication. The pharmacists were asked about their experiences regarding Turkish migrants’ and citizens’ attitudes and expectations, communication and contact with pharmacists and what would improve this communication. The Turkish citizens in Turkey and Turkish migrants were asked about knowledge, attitudes, decision making and behaviors regarding antibiotic use, as well as about ways of obtaining antibiotics and sources of information for antibiotic use.

In the three EU countries, focus group discussions with Turkish migrants were held in their native language and interpreters were present. Interviews were tape-recorded and transcribed. In one country (the Netherlands), eight in-depth interviews were held instead. All interviews were performed by researchers experienced in qualitative studies and interview methodology.

The interview data were analysed with conventional inductive content analysis in several steps [15]. First, the contents of the interviews with each target group were coded, assessed, categorised and documented in each country, supported with illustrative quotations. The coding procedure was inductive, identifying each quotation relevant for the research question. The coding and categorisation were developed and discussed by the research team in each of the countries (as listed in the author contribution list below).

Second, the information from the different target groups was synthesised and three relevant themes were identified. Furthermore, 11 categories were derived from the interviews in Turkey, and 11 categories from the corresponding data from the three EU countries.

The content of each theme was summarised in two sets, one for the data from Turkey, and one for the data from the three EU countries. The themes were jointly discussed and agreed upon by the researchers from each of the four countries.

The participants were informed about the study before agreeing to participate, and all participation was voluntary. No identification of the participants was registered and no data has been presented that can be connected to any identified individual.

## Results

### Implementation of regulations and recommendations in Turkey

Participants reported that there was *irrational antibiotic use among the public,* although it was now seen as *less likely to get antibiotics without a prescription* (Table 2). Compared with previous years, physicians were less likely to prescribe antibiotics on request from patients. Patient requests for antibiotics had also declined in recent years. However, there were indications of a variation between physicians, and family physicians were more likely to prescribe antibiotics, according to pharmacists. Pharmacists were less likely to sell antibiotics without a prescription, compared with the situation in the past.

*I believe physicians are now more careful about prescribing antibiotics than before. In the past, they used to prescribe antibiotics very frequently, even without thinking, but now they prescribe it very rarely. I think the Ministry of Health’s pressure on the physicians was effective (Citizen in Turkey)*

Citizens in Turkey *refraining from going to physicians and pharmacies* might have affected the potential for rational antibiotic use*.* Patients did not want to pay to visit a physician or did not have the time to do so. Pharmacists reported losing customers when applying a strict rule of not selling antibiotics without a prescription.

*I prefer not to go to a pharmacy and buy antibiotics without a prescription, because it is cheaper to buy antibiotic alone, then seeing a physician and paying fees both for the hospital visit and the medicine (Citizen in Turkey)*

Pharmacists believed that family physicians were now better educated and that the legal restrictions were effective. However, while *physicians were better educated, a lack of information remained among physicians*. Physicians reported not being informed about new policies and legislations in a timely and adequate way. Although physicians were not allowed to prescribe medicines that were not regulated within the social security system, they did not have electronic access to information about refund policies.

**Table 2:** Policy-relevant themes and categories concerning rational use of antibiotics in Turkey and among Turkish migrants in three EU countries: Germany, the Netherlands and Sweden

Themes	Implementation of regulations and recommendations		Access to antibiotics		Need for health communication	
Setting	Turkey	Turkish migrants in EU countries	Turkey	Turkish migrants in EU countries	Turkey	Turkish migrants in EU countries
**Categories**	Irrational antibiotic use among the public	Physicians were well-informed	Antibiotics could be obtained with or without a prescription	Access to antibiotics was mainly via prescription	Low level of knowledge about rational antibiotic use	Several sources of information
	Less likely to get antibiotics without a prescription	Few migrants got antibiotics without a prescription		Alternative ways to get antibiotics	Inadequate physician communication with patients	Communication with and trust in physicians were important
	Refraining from going to physicians and pharmacies	Pharmacists required prescriptions			Socioeconomic gradient in knowledge	Physician-patient communication could be improved
	Physicians were better educated, but a lack of information remained among physicians	Use of antibiotics was influenced by access to health care and medications			Interest in learning about antibiotics	Knowledge among migrants could be improved
	Lack of opportunities for physicians to evaluate patients adequately					Friends and families were of importance for habits
	Increased control of pharmacists					

There was also information about a *lack of opportunities for physicians to evaluate patients adequately* and manage care in accordance with present evidence. Physicians reported having a heavy workload and did not have time to evaluate patients adequately. Another problem for the family physicians in implementing rational use of antibiotics was that there was little opportunity for laboratory tests important for infection diagnostics in primary care settings.

Regarding the implementation of the new regulations on antibiotics, the interviews also showed that there was an *increased control of pharmacists* in Turkey*.* Pharmacists reported having been inspected by health authorities and warned by the inspectors not to sell antibiotics without a prescription.

### Implementation of regulations and recommendations as perceived by Turkish migrants in three EU countries

The respondents in the three EU countries reported that *physicians were well-informed* about rational antibiotic use (Table 2). Physicians were generally very conscious of when to prescribe antibiotics. Also, there were guidelines for rational use, and they were adopted by physicians and health care centres. Diagnostic tools for infection diagnostics were available and used by physicians, in order to facilitate rational use of antibiotics.

It was reported that *few migrants got antibiotics without a prescription*. Most migrants had similar attitudes and expectations as the general population.

The interviews showed that, generally, *pharmacists required prescriptions* for purchasing antibiotics in these countries. Pharmacists made it clear they would not provide antibiotics without a prescription, since it was illegal and could have consequences for the health and security of patients. Only a few migrants had asked for antibiotics without a prescription at pharmacies.

*It is very, very rarely that – in general with our patients, no matter where they are from – that they ask directly for antibiotics. By far, in most of the cases they just hand us a prescription from a doctor. And usually they don’t demand or expect a specific branded product (Pharmacist in Germany)*

*Use of antibiotics was influenced by access to health care and medications.* Antibiotics were paid for by health insurance companies, and were therefore more attractive than non-reimbursed medications. However, problems with getting in contact with physicians may have led to using alternative ways to get medicine.

### Access to antibiotics in Turkey

It was reported from citizens in Turkey that *antibiotics could be obtained with or without a prescription.* You could get antibiotics on prescription from a physician, with or without putting pressure on the physician (Table 2).

*I usually put pressure on the physicians and ask for antibiotics. If he or she resists, then I start questioning their medical knowledge and tell them they do not care enough about their patients (Citizen in Turkey)*

It was also possible to get antibiotics from pharmacies without a prescription, or from friends, family and neighbours.

*Some pharmacies sell antibiotics without a prescription. I know those pharmacies and go to those when I need antibiotics without prescription (Citizen in Turkey).*

### Access to antibiotics among Turkish migrants in three EU countries

The respondents stated that *access to antibiotics was mainly* via *prescription* (Table 2). Also, there was no difference in access to antibiotics between the migrant group and the majority population. Most of the respondents reported taking antibiotics on prescription, and the way it was prescribed by the physician.

However, there were *alternative ways to get antibiotics* for some Turkish migrants in the EU. Some got antibiotics in Turkey, also without a prescription, and brought them to the host country. It was also possible to get access to antibiotics online, without a prescription. Some migrants used leftover tablets from previous treatments, from family members or others. It was also reported that antibiotics were brought from the host country to relatives and friends in Turkey.

*I buy antibiotics when I travel to Turkey and use when I need and sometimes share them with my friends (Turkish migrant in Sweden)*

### Need for health communication in Turkey

The respondents reported that there was a *low level of knowledge about rational antibiotic use* among citizens in Turkey (Table 2)**.** Most participants were unaware of the negative consequences of irrational antibiotic use. The participants’ general knowledge of rational antibiotic use was also low.

*Antibiotics are used to treat illnesses in a very short time. They shorten the duration of the illnesses. For example, they are used to treat severe pain and high body temperature. For instance, I use them when I have a toothache (Citizen in Turkey)*

There were several reports about *inadequate physician communication with patients.* According to participants, physicians did not provide adequate information on how to use antibiotics. On the other hand, physicians reported providing (mainly verbal) information to patients, but those patients did not understand the information, or did not want to use medicine once they were feeling better.

*I think there is a big discrepancy between what physicians say. One says one thing and the other says something against it. We cannot decide whom to believe. I have many questions in mind, but there are different answers. I feel totally confused about medicines (Citizen in Turkey)*

There was an indication of a *socioeconomic gradient in knowledge* of rational antibiotic use in Turkey. The knowledge of rational antibiotic use was lower and requests for antibiotics were more common among lower socioeconomic groups. However, in general, the study participants showed an *interest in learning about antibiotics* both from physicians and from different media.

### Need for health communication among Turkish migrants in the three EU countries

Turkish migrants in the three EU countries reported using *several sources of information* for learning more about antibiotics (Table 1). Most participants got information about antibiotics from their physician or pharmacist. Turkish migrants often asked pharmacists questions about antibiotics. The manufacturers’ leaflets were an important additional source of information in the migrant group. Younger participants also mentioned the internet as an information source.

The respondents believed that *communication with and trust in physicians were important* for appropriate use of antibiotics. The patients’ trust in the physician was a key factor for any treatment decision. Physicians often asked patients about their concerns and expectations and tried to reach an agreement with the patient on how to manage the health problem.

There were some comments indicating that the *physician-patient communication could be improved.* For instance, Turkish migrants generally did not take an active patient role at consultations. Some participants had little trust in physicians in the host country and criticised them for not prescribing medications.

*I had to recover naturally and had to come back in a few days. I felt so much worse and then, finally, got my antibiotics prescribed. I accepted what my GP says and waited for one week. I was not satisfied, however (Turkish migrant in the Netherlands)*

Information from physicians and pharmacies was mainly verbal. There was a lack of written information, especially in the migrants’ mother tongue language. Findings indicated that there might be a language barrier in verbal communication. Another limitation mentioned was that the information from physicians often focused on instructions for administration of medicine and side effects, but not on the broader concept of rational use.

There were also indications that the *knowledge among migrants could be improved*. Some migrants knew that antibiotics were only effective for bacterial infections, while some believed antibiotics were effective against catching a cold and influenza. In addition, migrants often stopped taking antibiotics when they were free of symptoms.

*Friends and families were of importance for habits*, according to information from the respondents. Mothers were often responsible for managing medications within families. Positive attitudes to antibiotics might have been due to prior positive experiences, including personal use, but also the experiences of close friends, relatives and other trusted people.

## Discussion

Among participants in Turkey, it was generally considered less likely to get antibiotics without a prescription now than in the past. Patient requests for antibiotics had declined, physicians were believed to be better educated and regulations were considered to be effective. However, several barriers at the health system level were identified. Physicians still lacked access to information on new policies and rules. High workloads and a lack of laboratory facilities also limited the opportunities for physicians to deliver evidence-based care. Among pharmacists in Turkey, where the pharmacy sector was privatised at the time of the study, there was a fear of losing customers if they applied the rules strictly, and patients were not believed to be willing to pay to visit physicians if they did not get medications.

In the three EU countries studied, it was more established that pharmacists would require a prescription to dispense medication and physicians were better educated. Most Turkish migrants had adopted the patterns of rational antibiotic use common in these countries. In addition, there were health system factors that were reported to influence the use of antibiotics in these countries. For instance, problems with getting in contact with physicians could lead to the choice of other alternatives to get treatment for infections. It was reported that health insurance companies readily paid for antibiotics, which might increase demand for these drugs.

Previous research has analysed factors that influence self-medication with antibiotics, mainly patient factors, while there are fewer studies which focused on the health system level [13], although a lack of rapid and sufficient diagnostic tests has been discussed [18]. This comparative study contributes by illustrating the variation in the opportunities for rational drug use in different health systems.

Despite new regulations and an increased awareness of rational use, it was still possible to get access to antibiotics in alternative ways in Turkey, for instance by requesting a prescription from a physician. It was said to be possible to find a pharmacy that would sell antibiotics even if the customer did not have a prescription. For Turkish migrants in the EU countries, one alternative was to travel to Turkey to get medications, or to get them from friends and relatives in Turkey. These migration-related self-medication alternatives have very rarely been explored in the literature, but they were clearly identified in the present study. Since AMR is a global threat, there should be more focus on between-country variations in systems and their consequences for migrants. There is a need for harmonising regulations, policies and strategies between countries, in order to facilitate rational use.

The study also illustrates the need for high-quality health communication in the field of rational use of antibiotics. Although a new national programme has been initiated in Turkey, there are still indications of low public knowledge regarding antibiotics. There were reports of poor physician-patient communication in this field, and a lack of information sources in Turkey. There were also reports of a socioeconomic gradient in the knowledge of rational use. In the EU countries, migrants reported having access to a broader range of information sources. Still, several migrants reported communication problems and problems in accessing written information in their native language, and there were signs of a lack of knowledge. Thus, health literacy seemed to be limited. The need for patient education and qualitative physician-patient communication has been highlighted before [13, 18]. Strategies to improve rational use often include knowledge dissemination as one of the major tools [4].

We believe methods for health communication and patient-centred care should be developed and implemented on a broader scale, in order to achieve the programme goals. One important aspect is to develop qualitative communication skills to manage differing levels of health literacy between population groups [19].

This paper is based on qualitative individual interviews and focus group discussions. We have explored the perceptions, among the public, physicians and pharmacists, of factors influencing rational antibiotic use. The study cannot be conclusive when it comes to what causes irrational medication. However, the study is based on interviews with a total of 130 respondents in four countries, and brings new insights through the inclusion of comparisons between health systems, as well as a migrant perspective. We believe that the study adds knowledge on the issues of promoting rational antibiotic use in Turkey and EU countries, as well as on the issue of migrant-friendly health services in host countries.

## Conclusions

We have found the following main policy implications:

- There is a need for harmonisation of health-related regulations and policy programmes.
- Antibiotics should only be available with a physician’s prescription, and legislation on cross-border and internet-based sales should be adapted to this.
- Programmes for promoting rational antibiotic use need to be implemented on a broad scale, in medical care, at pharmacies and in the population.
- Educational programmes should include goals to increase general health literacy and specific knowledge about the rational antibiotic use.
- Methods for health communication and patient-centred care should be developed and implemented in this field.

## Data Availability

The datasets used in the current study are not publicly available due to integrity statements in the ethical approvals.
